# FEASIBILITY AND EFFICACY OF LIFE REVIEW DEPRESSION INTERVENTION BY VIDEO-TRAINED FAMILY CAREGIVERS

**DOI:** 10.1093/geroni/igad104.0281

**Published:** 2023-12-21

**Authors:** Christina Miyawaki, Angela McClellan, Erin Bouldin, Cheryl Brohard, Mark Kunik

**Affiliations:** University of Houston, Houston, Texas, United States; Baylor University, Waco, Texas, United States; University of Utah, Salt Lake City, Utah, United States; University of Houston, Houston, Texas, United States; Baylor College of Medicine, Houston, Texas, United States

## Abstract

Due to the high prevalence of depressive symptoms and dementia in older Americans, we developed a six-week virtual depression intervention, Caregiver-Provided Life Review (C-PLR) for care recipients (CRs) with early-stage dementia and mild depression and their family caregivers (CGs). We changed the length of CG training iteratively, trained CGs in interviewing and probing skills via video, and CGs delivered life reviews (LR) with CRs at home (N=25 dyads). The objective of the study was to examine the feasibility and efficacy of C-PLR on CRs who were community-dwellers and long-term care facility residents. We collected pre- and post-measures of CRs’ depression (primary outcome) and life satisfaction, CGs’ burden, positive aspects of caregiving, and CG-CR relationship quality (secondary outcomes) and compared them using paired t-tests. We evaluated if the effect differed by race/ethnicity, residential setting, or living arrangements. CGs were 52 years old (mean), working (64%), college-educated (72%), female (88%), and in good-excellent health (72%). CRs were 81 years old (mean), female (84%), and in poor-fair health (56%). CRs’ depression significantly improved following C-PLR (p< 0.001), and this effect was found in CRs who were Asian (p=0.017), White (p=0.040), community-dwelling (p< 0.001), lived alone (p=0.045), and lived with others (p=0.002). Secondary outcomes did not change. This study demonstrated that C-PLR can be successfully taught to CGs via video and effectively reduces CRs’ depressive symptoms without increasing CG burden. C-PLR could be implemented broadly to improve symptoms among CRs in community and residential settings, as well as among a diverse population of CRs.

